# Supplementary material for: Challenges and opportunities for integrating traditional healing approaches with biomedical care for mental illness: A scoping review from healers’ perspectives
Source: PLoS One. 2025 Jun 3;20(6):e0325353. doi: 10.1371/journal.pone.0325353 (PMC12132947; doi:10.1371/journal.pone.0325353)
Supplement: Attachment — Submitted filename: review comments.docx [file pone.0325353.s002.docx]

**Title -** Integrating Biomedical Care with Traditional Healing vs Integrating Traditional Treatment Approaches with Biomedical Treatment, which sounds and is described differently in the document. Does it have differences? In addition, integration and collaboration terms are different in health systems, therefore, it needs operation definition in this research.

**Background:**

- it is good to add existing evidence related to integrated services effectiveness, the challenges and its opportunities and consider the word limit of the journal. as, there is no compiled evidence on the challenges and opportunities related to integrating traditional healing practices with modern mental health care from the healers’ perspectives. This statement seems very general as the evidence which may be not adequate and your research gap filling should be clearly addressed.
- Do you think the global context has similar integration challenges and opportunities? I recommend addressing this issue in different context such as European context, Asian , Africa etc as background content . then, it can be help during the discussion and thematic result writing process.

**Methodology**:

- What was the reason for the search was limited to January 2014 and June 30, 2024?
- Was the list of synonyms or alternative terms developed for the initial keywords for the search strategy?
- Was it done manually? After removing duplicates and filtering important articles, the data were extracted using the prepared data extraction form
- Would describe clearly about thematizing process including the coding process example was it deductive or inductive, was it coded only by one person? Use reference for the thematic analysis as there are different steps and guides of thematic framework analysis.
- Do you use any tool to assure the quality of the included articles?
- Describe the analysis process to assure the trustworthiness meaning the validity of quality of the research data. Example, good to include the annex of Nvivo exported codes indexation and make clear if the coding process was done by independent reviewers.

**Result:**

- **Under the review of studies**. It is described that three review articles are included. It is not clear that if all articles are included in your study, why the existing review studies included that means the single articles finding extracted data, again be extracted their finding indirectly from the other reviews citation(if included). In addition, if that is the case, the main significance of this review in comparison to previously published reviews needs to be clearly justified. Could you elaborate on this point to clarify the unique contribution and added value of your review?
- **Attitude challenges related to health professionals** …“Emenet,” what is does mean ?
- **Attitude challenges related to health professionals** line 20 - biomedical staff Vs , under **Attitudinal challenges** of healers line 2 - western practitioners ….. would you make it consistent through all the documents in using such terms?
- The result writing flows to make it easy for readers, I prefer to make it starting from developed to developing or something else flow. some paragraphs start to state the result findings from Africa to UAE and then to Africa. if the statements are not interconnected, I prefer stating the findings with similar contexts.
- **Attitudes of service users, line 2** - Some patients and caregivers are reluctant…. Are studies among such participants (patients and caregivers) included in your review?
- Policy issues – references to the articles are not included.

**Discussion**

- The repetition of similar **subtopics** in the result and in the discussion including repeating results of some paragraphs in the discussion are already in the way they are written in the result … this makes it boring for readers and better to consider a revision.
